# Acute-on-chronic liver failure: a retrospective review of cases at a transplantation center in Brazil

**DOI:** 10.1590/acb392624

**Published:** 2024-06-14

**Authors:** Fernando Camargo Fernandes, Amanda Pinter Carvalheiro da Silva Boteon, Giovana Garcia Rossi, Fernanda Marques, Bianca Della-Guardia, Yuri Longatto Boteon

**Affiliations:** 1 Faculdade Israelita de Ciências da Saúde Albert Einstein – São Paulo (SP) – Brazil.; 2 Hospital Israelita Albert Einstein – São Paulo (SP) – Brazil.

**Keywords:** Liver Transplantation, Cirrhosis, Acute-On-Chronic Liver Failure, End Stage Liver Disease

## Abstract

**Purpose:**

Acute-on-chronic liver failure (ACLF) is a leading cause of death in cirrhotic patients. This study aims to describe the outcomes of in-patients with ACLF at a liver transplantation (LT) center in Brazil.

**Methods:**

Retrospective study analyzing patient data from 2017 to 2022. Re-transplant cases and patients without previous chronic liver disease were excluded. The ACLF diagnosis was based on the European Association for the Study of the Liver-Chronic Liver Failure criteria and assessments repeated on days 3 and 7 after the initial diagnosis.

**Results:**

Among 381 patients, 10.49% (n = 40) were diagnosed with ACLF. Bacterial infection was the most common precipitating factor (45%). Kidney failure occurred in 65% of the cases. The 28-day mortality rate was 35% and varied according to ACLF severity at diagnosis, from single organ failure (ACLF-1) at 22% to three organ failures (ACLF-3) at 60%. Eighteen patients (45%) were transplanted with a 100% 28-day survival rate. For ACLF-3 cases at diagnosis (n = 15), the 28-day and 1-year survival rates with a transplant (n = 4) were 100% and 80%, respectively, and without transplant (n = 11), 10 and 0%, respectively.

**Conclusions:**

ACLF was associated with high mortality rates. LT was an effective therapeutic option, particularly for ACLF-3 cases.

## Introduction

Acute-on-chronic liver failure (ACLF) is a sudden deterioration of pre-existing chronic liver disease, typically related to a precipitating event^1^^-^ 3. It is currently one of the leading causes of death among patients with cirrhosis who are on a waiting list for a liver transplantation^1^^,^ 3 ^,^ 4. Although early transplantation is a treatment option for selected ACLF patients, previous studies have indicated that this approach can be ineffective for the most severe ACLF cases^3^^,^ 5 ^,^ 6. Therefore, further data on the global epidemiology of this condition, the outcomes for affected patients, its effect on the number of transplantations, and its early identification are crucial to ensure adequate and timely interventions^6^^,^ 7.

While there is currently no precise definition of ACLF, it is a consensus that these patients have a poor prognosis^1^^,^ 8 ^,^ 9. The European Association for the Study of the Liver-Chronic Liver Failure (EASL-CLIF) Consortium considers ACLF a specific syndrome in cirrhotic patients characterized by acute decompensation, organ failure and high short-term mortality^1^^,^ 10 ^,^ 11. In addition, through the Acute-on-Chronic Liver Failure in Cirrhosis (CANONIC) study, the EASL-CLIF Consortium classified ACLF using the CLIF-Sequential Organ Failure Assessment (CLIF-C SOFA) scoring system, which is a modification of the original SOFA score^1^^,^ 11.

In a single-center Brazilian study, an ACLF prevalence of 37% was found among cirrhotic patients admitted to a hospital emergency unit with decompensated cirrhosis. The 28-day survival rate for these patients was 38.9% 12. A previous Brazilian study, with a similar design, reported ACLF rates of 24% with a 30-day mortality frequency of 35%^13^. The Prevalence, Epidemiology, Characterization and Mechanisms of ACLF in Latin America (ACLARA) study was a large, prospective, observational investigation focusing on the correlation between genetic ancestry, race, and ACLF. The study included a large patient sample from 12 Brazilian centers and reported a 35% prevalence rate of ACLF^14^. Despite having descriptive data, there is a lack of detailed information in the national literature regarding the prevalence, treatment options, and outcomes of ACLF patients in transplant centers in Brazil. The frequency of liver transplants and patient outcomes for this severe condition is largely unknown in developing countries due to limited data availability. The only exception is the ACLARA study, which included only 66 transplant cases among all centers in Latin America. Nevertheless, the outcomes of patients at individual centers or countries are not described. This investigation is particularly relevant, given the findings of the latter research indicating that an increasing percentage of Native American genetic ancestry and race (vs European American race) were independently associated with increased odds of ACLF^14^.

Therefore, this study aimed to describe the characteristics and outcomes of in-patients with ACLF at a liver transplant center in Brazil. It also analyzed precipitating factors for ACLF, patient survival, and the frequency of transplants.

## Methods

### Study design

This retrospective observational study reviewed medical data from electronic health records of patients assessed in the liver transplant program at Hospital Israelita Albert Einstein in São Paulo, Brazil, from June 2017 to June 2022. The study protocol complied with the ethical guidelines of the Declaration of Helsinki. It was approved by the Research Ethics Committee of the Hospital Israelita Albert Einstein in accordance with the National Health Council´s resolution 466 of 2012 (approval number 5.637.066), with an exemption from the informed consent requirement. This study was reported under the Strengthening the Reporting of Observational Studies in Epidemiology (STROBE) initiative statement.

### Participants

Patients with cirrhosis and ACLF per EASL-CLIF criteria, evaluated for or listed for liver transplantation prior to admission or referred for urgent transplant evaluation, over 18 years old, were eligible for the study. The diagnosis of cirrhosis was defined by liver biopsy or a combination of clinical assessment, imaging and laboratory findings. All in-patients admitted to the intensive care unit (ICU) and those admitted to the ward before receiving a transplant who had acute decompensation with liver failure (total bilirubin > 12 mg/dL) or coagulation failure [international normalized ratio (INR) ≥ 2.5] or developed this condition, were investigated for the occurrence of organ failure according to the EASL-CLIF Consortium criteria.

Patients without previous chronic liver disease, re-transplant cases, complications of other severe, chronic, extra-hepatic diseases or positive for human immunodeficiency virus infection were excluded from the analysis. For patients with more than one hospital admission, all ACLF assessments were considered. In cases where an ACLF diagnosis resulted in more than one admission, the first ACLF assessment was included in the analysis.

### Analyzed outcomes

Data on patient outcomes, including the occurrence of ACLF, 28-day, 90-day, and 1-year mortality rates post-diagnosis, transplant status, and survival at the last follow-up, were collected. Mortality during the study period was defined as death from any cause within 1 year of the diagnosis of ACLF. Patient follow-up started on the date of the ACLF diagnosis, and patients were followed until death, the last follow-up date or one year after diagnosis.

### ACLF diagnostic criteria and stratification

The EASL-CLIF Consortium criteria for organ failure used to define ACLF are: *i*) liver: total bilirubin ≥ 12 mg/dL; *ii*) kidney: creatinine ≥ 2 mg/dL or use of renal replacement therapy; *iii*) coagulation: INR ≥ 2.5; *iv*) brain: hepatic encephalopathy grades 3–4 using the West Haven classification or requiring mechanical ventilation; *v*) circulation: use of vasopressors, including terlipressin; and vi) lung: the ratio of partial pressure of arterial oxygen to the fraction of inspiratory oxygen concentration (PaO_2_/FiO_2_) ≤ 200 or oxygen saturation (SpO_2_)/FiO_2_ ≤ 214, or use of mechanical ventilation for a reason other than hepatic encephalopathy.

ACLF was classified into three degrees of severity. ACLF grade 1 has three sub-groups, which include: single kidney failure; single liver, coagulation, circulatory or lung failure associated with a serum creatinine level ranging from 1.5 to 1.9 mg/dL, mild to moderate hepatic encephalopathy, or both; and single brain failure and serum creatinine level ranging from 1.5 to 1.9 mg/dL. ACLF grade 2 involves two organ failures, while ACLF grade 3 is characterized by three or more organ failures.

The presence of ACLF and its grade assessment was repeated on days 3 and 7 after the initial diagnosis.

### Data collection and study variables

This study used secondary and retrospective data previously anonymized to researchers by the Liver Transplant Program Management Team at the Hospital Israelita Albert Einstein. Patient demographic and clinical data included the etiology of liver disease, the Model for End-Stage Liver Disease (MELD) scores at transplant, the occurrence of hepatocellular carcinoma, whether there was a need for a re-transplant, mortality, the presence of hepatic encephalopathy according to the West Haven classification, the use of vasopressors (terlipressin or catecholamines), mean arterial pressure, whether there was a need for mechanical ventilation, the FiO_2_ and SpO_2_. The following precipitating factors for ACLF development were examined: exacerbation of hepatitis B, bacterial infection, gastrointestinal bleeding, active alcoholism in the last three months, others (trans-jugular intra-hepatic portosystemic shunt, surgery, large-volume paracentesis without albumin, hepatitis, alcoholic hepatitis), unidentifiable causes and situations where there was more than one cause. The reviewed laboratory tests included the INR, creatinine levels, serum total bilirubin and PaO_2_.

### Liver transplants for ACLF patients

The Brazilian Ministry of Health regulations employ the MELD system for donor organ allocation in liver transplantation. There is no prioritization of transplant patients with ACLF. In addition, no national or local policy precludes liver transplantations for this population. Therefore, this procedure is considered for every patient with a clinical condition that may be treated with a transplant. Although there were no fixed criteria, there was local consensus at the hospital that, to prevent possible transplantation failures, transplant would not be considered for patients that needed two vasoactive drugs, those with raised lactate levels (> 4 mmol/L), those in septic shock, or patients with multi-drug-resistant infections.

### Statistical analysis

Categorical variables were described and analyzed using absolute frequencies and percentages and compared using Pearson’s chi-square or Fisher’s exact test. Multiple comparisons of significant results were conducted using the Benjamini-Hochberg p-value adjustment method. Continuous variables were described using means and standard deviations or medians and quartiles and minimum and maximum values (normality was evaluated using the Shapiro-Wilk test) and analyzed using the Student’s T-test, the Mann-Whitney test, analysis of variance, or the Kruskal-Wallis test, depending on the distribution and number of comparison groups. Survival was estimated using Kaplan-Meier plots with log-rank tests to compare differences in survival curves between groups. The effect of covariates on the failure rate of patients was investigated using univariable and multivariable Cox regression models. Appropriate techniques for diagnosing the assumptions of the Cox model were applied. The significance level was p ≤ 0.05. All analyses were conducted using R Statistical Software (v4.1.1; R Core Team, 2021).

## Results

### Prevalence and severity of ACLF at enrolment

**Figure 1:**
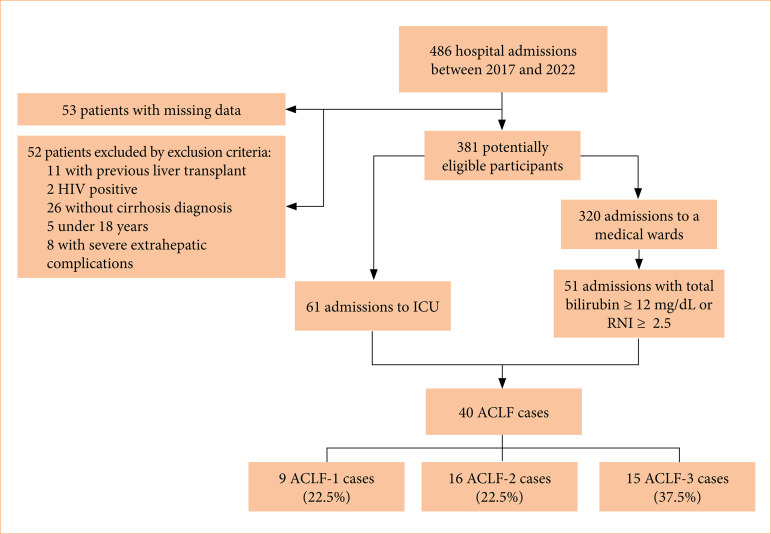
Flowchart of patient study inclusion to identify acute-on-chronic liver failure cases.

HIV: Human Immunodeficiency Virus; ICU: Intensive care unit; ACLF: Acute-on-chronic liver failure; ACLF-1: single organ failure; ACLF-2: two organ failures; ACLF-3: three organ failures.

Data from 486 patients admitted during the study period were reviewed. After excluding cases with incomplete data, the liver or coagulation failure criteria were applied together with the exclusion criteria to screen for ACLF cases. Of the 381 eligible patients, 40 cases of ACLF (10.49%) were included in the study. Figure 1 presents a patient inclusion flowchart. In regard to the severity of ACLF at presentation, 9 patients (22.5%) had ACLF grade 1, 16 (40%) presented with grade 2, and 15 (37.5%) had grade 3.

### Clinical characteristics and factors associated with ACLF

The mean patient age was 51 years, and 21 patients (52.5%) were female. Subjects were predominantly admitted to the ICU (82.5%, 33 cases). The most common etiology underlying chronic liver disease was combined causes (22.5%), followed by alcohol abuse (17.5%), metabolic-associated steatohepatitis (15%) and autoimmune hepatitis (15%). Four cases (10%) were found to have hepatocellular carcinoma. Infection was the most common precipitating factor (45%) for ACLF, primarily spontaneous bacterial peritonitis, pneumonia, urinary tract infection, and skin infections. The most frequent organ to fail was the kidney (65%), followed by the liver (52.5%) and coagulation (37.5%). The initial clinical characteristics of the patients are listed in Table 1.

**Table 1:** Baseline patient characteristics, precipitating factors, and frequency of organ failure due to acute-on-chronic liver failure.

Demographic, clinical and laboratory data		All (n = 40)
Age (years), mean ± SD		51 ± 15
Male, n (%)		19 (47.5)
Etiology of cirrhosis, n (%)		
	Alcohol	7 (17.5)
	MASH	6 (15)
	Hepatitis B	4 (10)
	Hepatitis C	2 (5)
	Autoimmune hepatitis	6 (15)
	Cryptogenic	4 (10)
	Primary sclerosing cholangitis	3 (7.5)
	Combined causes	8 (22.5)
Precipitating factor, n (%)		
	Bacterial infection	18 (45)
	Hepatitis B exacerbation	2 (5)
	Gastrointestinal bleeding	5 (1255)
	Others	9 (22.5)
	More than one factor	1 (2.5)
	Undetected	5 (12.5)
Organ failures, n (%)		
	Liver	21 (52.5)
	Coagulation	15 (37.5)
	Kidney	26 (65)
	Circulation	14 (35)
	Lungs	7 (17.5)
	Cerebral	5 (12.5)
Laboratory data		
	Total bilirubin (mg/dL), median (IQR)	11.9 (1.87 – 28.6)
	Serum creatinine (mg/dL), median (IQR)	2.2 (1.28 – 2.91)
	INR, median (IQR)	2.15 (1.71 – 3.16)

Abbreviations: MELD – Model for End-Stage Liver Disease; MASH – Metabolic associated steatohepatitis; IQR – interquartile range, quartiles 1 and 3.

### Evolving aspects of ACLF cases after diagnosis

For 19 cases (47.5%), the initial ACLF severity classification remained stable after three days; for 11 patients (27.5%), it remained stable after seven days. For 9 and 19 patients on days 3 and 7, respectively, reassessments were not made due to either having received a transplant or death (Table 2).

**Table 2:** Clinical course of acute-on-chronic liver failure cases on days 3 and 7 from diagnosis.

Period		n (%)
From diagnosis to day 3 (n = 31), n (%)		
	Improved	5 (12.5)
	Worsened	7 (17.5)
	Remained stable	19 (47.5)
From diagnosis to day 7 (n = 21), n (%)		
	Improved	6 (15)
	Worsened	4 (10)
	Remained stable	11 (27.5)

### Clinical outcomes of ACLF cases

Regarding the overall clinical outcome of the patients, 15 (37.5 %) died, 18 (45%) received transplants, and 7 (17.5%) recovered without a transplant. There was a statistically significant difference between patients’ global outcomes according to their ACLF initial severity. While the most frequent outcome for patients with ACLF grades 1 and 2 was receiving a transplant, death was the most common outcome for patients with ACLF grade 3 (Chi-Square test, p = 0.021). The outcomes of the ACLF case are presented in Table 3.

**Table 3:** Prognosis of acute-on-chronic liver failure cases according to the number of organ failures by the European Association for the Study of the Liver-Chronic Liver Failure (EASL-CLIF) definition.

Variable	ACLF grade at presentation, n (%)			p
	1	2	3	
Final ACLF outcome*				
Death	2 (22)	3 (19)	10 (67)	0.021
Transplant	4 (44)	9 (56)	5 (33)	
Resolution	3 (33)	4 (25)	0 (0)	
28-day mortality	2 (22)	3 (19)	9 (60)	0.054
90-day mortality	3 (33)	6 (38)	11 (73)	0.082
1-year mortality	7 (78)	6 (38)	11 (73)	0.066

ACLF: Acute-on-chronic Liver Failure.

* Chi-Square test (5% significance level).

### Survival analysis of ACLF patients

**Figure 2:**
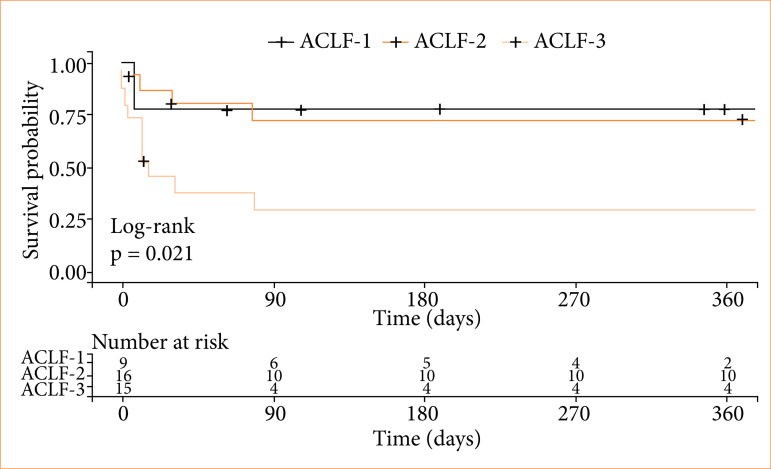
Patient survival by acute-on-chronic liver failure severity at diagnosis.

ACLF: Acute-on-chronic liver failure; ACLF-1: single organ failure; ACLF-2: two organ failures; ACLF-3: three organ failures.

The overall 28-day mortality rate was 35% and exhibited an increasing trend of differing mortality according to the severity of ACLF at the time of diagnosis (p = 0.054), with ACLF grade 1 at 22% mortality rate and ACLF grade 3 at 60%. This trend was also found for 90-day (p = 0.082) and 1-year mortality rates (p = 0.066) (Table 3). These findings were confirmed by the overall patient survival analysis (which applied the log-rank test to the Kaplan-Meier Curve), which found a significant difference according to the severity of ACLF at the time of diagnosis (p = 0.021) (Fig. 2). In the univariate Cox logistic regression analysis, ACLF grade 3 at presentation was significantly associated with a 3.8-fold increased risk of mortality (hazard ratio = 3.864, 95% confidence interval 1.394–10.71, p = 0.009) when compared with grades 1 and 2 combined.

### Liver transplants for ACLF patients

Eighteen patients (45%) were transplanted within a median of 6 days (first and third quartiles 2.5–10.75; minimum 0; maximum 27). The mean age at transplant was 45.88 years (standard deviation 18.96; minimum 18; maximum 72), and the MELD score was 34.41 (standard deviation 7.43; minimum 25; maximum 48). The patients’ 28-day and 1-year survival rates were 100% and 83%, respectively. Re-transplantation was necessary for two patients.

For the ACLF grade 3 cases at presentation (n = 15), the 28-day and 1-year survival rate after receiving a transplant (n = 4) was 100% and 80%, and without a transplant (n = 11), 10% and 0%, respectively.

## Discussion

ACLF is a condition that affects patients with chronic liver disease and is characterized by organ failure and high mortality^1^. Surveillance for its occurrence is crucial for in-patients admitted to hospital with acute decompensation of cirrhosis due to the risk of rapid worsening of the condition, death and the need to consider a liver transplantation within a short time — especially for ACLF grade 3 cases. The screening process should primarily focus on cirrhotic patients admitted to the ICU, especially those affected by infectious diseases or who have developed renal dysfunction. In our study, ACLF grade 3 was an independent risk factor for mortality. However, receiving a liver transplantation resulted in excellent survival results, proving tot be a life-saving procedure, especially in this most threatened population.

The prevalence of ACLF varies between studies according to the diagnostic criteria used and the population under investigation. In the CANONIC study, 22.6% of the 1,343 patients evaluated were diagnosed with ACLF 1. In another study, which applied the Asia Pacific Association for the Study of the Liver (APASL) criteria, ACLF was diagnosed in 12% of patients admitted to hospital for complicated cirrhosis^15^. In Brazil, two studies using the EASL-CLIF criteria found an ACLF prevalence of 35.3% in 2019 and 24% in 2015^12^^,^ 13. Both studies were conducted in secondary care hospitals, not transplants reference centers, and evaluated only a limited number of patients. In this study, the frequency of ACLF was 10.49%, a rate significantly lower than previously described for Brazil. This finding may be a consequence of having been conducted in a tertiary center and regional/national reference hospital for organ transplants that primarily included patients previously listed or under evaluation for the procedure or those without legal contraindications (e.g. active alcoholism) with a request for an urgent transplant assessment. Besides, all patients admitted to the liver transplant unit were screened, regardless of suspected decompensation of cirrhosis. Furthermore, and contrary to the two other Brazilian studies^12^^,^ 13 and the CANONIC study in which ACLF grade 1 cases were predominant^1^, in our sample, there was a predominance of more advanced degrees of ACLF — 40% with ACLF grade 2 and 37.5% with ACLF grade 3.

The mortality rate of ACLF patients in our study was 35% over a 28-day period, similar to values reported in the literature^3^^,^ 6. This value is also similar to the previous 2019 national study (39%)^12^. However, it is well below that reported by the 2015 Brazilian study (65%)^13^. This finding may be due to developments in ACLF supportive treatment and local conditions at the center. However, it is more likely related to the risk factors for mortality identified in the latter study, such as active alcoholism (p = 0.041) and other decompensations of cirrhosis^13^. It should also be noted that the 28-day mortality rate in our study increased in proportion to the number of organ failures, varying from grades 1 to 3, from 22 to 60%, values similar to those reported in the literature for the EASL-CLIF criteria^3^^,^ 6. However, the mortality rate in our study for ACLF grade 3 patients was well below that described in the two Brazilian studies mentioned^12^^,^ 13, which recoded 100% of mortality. The reason for this could be attributed to the high probability of patients undergoing a transplant in a brief period during our research..

Aside from receiving a transplant, ACLF treatment is based on diagnosing and treating the precipitating factors of decompensation and supportive therapy for organ dysfunction. Bacterial infections were the most common precipitating factor for ACLF in our sample, which agrees with reports from European, Asian and North American countries^7^^,^ 16. Thus, it reinforces the need for infectious disease screening in this population at minimal suspicion of a diagnosis. However, despite the importance of identifying precipitating factors, they remain undetermined in up to 1/3 of cases^10^^,^ 17.

In a recent systematic literature review and meta-analysis, Mezzano et al. found renal dysfunction the most common organ failure (49%) in ACLF patients^7^. This is consistent with our study’s findings (65%). Therefore, 85.3% of the cases in our study required admission to the ICU for supportive treatment of organ failure. The CANONIC study found that only isolated acute kidney injury was associated with a mortality rate greater than 15% at 28 days^1^, a higher rate than for other organ dysfunctions alone, justifying its classification as ACLF grade 1 — which is not the case for other isolated organ failures.

In addition to supportive therapy for organ failure, ACLF is a dynamic syndrome requiring continuous monitoring of patients with ACLF due to the risk of rapid changes in a patient’s clinical condition. Within three days of diagnosis (and in less than half of the patients studied), the severity of the condition remained stable, and within the group where conditions changed, only 12.5% improved. When the interval from diagnosis to day 7 of follow-up was considered, the data showed even greater disparity. Furthermore, the initial severity of ACLF, accessed through the number of organ failures according to the EASL-CLIF definition, was significantly associated with patient outcome. The 28-day mortality rate in our study ranged from 22% for ACLF grade 1 to 60% for ACLF grade 3. The mortality rate for ACLF grade 3 patients in our study was lower than previously described, reaching 80%^3^, most likely due to the possibility of receiving a timely liver transplant as a therapeutic alternative. Due to the limited number of cases and the rapid loss of the sample due to either receiving a transplant or death, it was not possible to estimate the effect of the clinical course of ACLF on patient outcomes 3 to 7 days after diagnosis — a factor also associated with unfavorable outcomes^18^^-^ 20.

Liver transplantation proved to be a life-saving therapy for ACLF patients, especially for grade 3 cases. At this most severe stage of the condition at diagnosis, the 28-day and 1-year survival rates with a transplant corresponded to 100 and 80%, respectively. Without a transplant, the survival rates were 10% and 0%, respectively. These values are similar to international transplant centers, which have reported 1-year survival rates for this population with and without transplants of 83.9 and 7.9%, respectively 21 ^,^ 22. The higher survival rates in this study can be attributed to the fact that the subjects were often already listed for a transplant. This enabled their prompt activation in the waiting list within an adequate therapeutic window. However, the effect of other variables that affect transplant outcomes deserves detailed investigation, such as the type of donor organ used (e.g. an extended criteria donor or living donor liver transplant) and the possibility of using new technologies for preserving organs for transplants^23^^,^ 24.

The present study has some limitations. One of them is that it is a single-center retrospective study carried out in a tertiary hospital that is a reference in health care in the Brazilian scenario. Thus, the available resources and clinical assistance represented a cross-sectional portrait of the study period and differed from the typical parameters seen in health centers in Brazil. Therefore, caution should be taken when generalizing our results to other locations and developing countries, as health systems and patient characteristics may vary considerably. Furthermore, a cut-off value for laboratory parameters (total bilirubin > 12 mg/dL and INR ≥ 2.5) was used as a screening tool for liver or coagulation failures and to initiate a complete assessment of the EASL-CLFI ACLF diagnostic criteria for patients admitted to the hospital. This means that, although unlikely, there may have been patients with ACLF who were not considered in the study sample. Furthermore, the small number of ACLF cases studied and the loss of patients – either due to death for the most severe stages of the disease or to follow-up for the less severe cases – made it challenging to compare survival rates for individual ACLF groups by disease severity. However, even with these limitations, when comparing the survival curves of the groups categorized as ACLF grades 1 and 2 to ACLF grade 3, the latter showed a significantly higher mortality rate, consistent with a more severe clinical condition. The retrospective study design using existing medical data from electronic health records, may limit the variables for analysis, which may impact the results. Finally, due to an electronic medical record system change during the study period, data for 53 patients in the transplant program after hospital admission was lost. which prevented the inclusion of these cases. In the future, national multi-center studies may create a more reliable picture of the treatment situation and clinical outcomes of patients with ACLF in Brazil. It may also encourage the discussion of possible prioritization criteria for receiving a transplant for patients with this severe condition.

## Conclusion

In our cohort, ACLF was associated with a high mortality rate, and infectious diseases were the main precipitating factor for organ decompensation in these patients. When comparing the survival curves of ACLF grades 1 and 2 to ACLF grade 3, the latter had a significantly higher mortality rate. A timely liver transplant was an efficient therapeutic option that significantly increased patient survival, especially for those affected by a greater number of organ dysfunctions.

## Data Availability

All data sets were generated or analyzed in the current study.
